# Athlete-specific force asymmetry monitoring in collegiate volleyball: a prospective observational cohort

**DOI:** 10.3389/fspor.2026.1885824

**Published:** 2026-08-04

**Authors:** Nicholas M. D’Ambrose, Dana H. Tran, Mitchell J. Christiansen, Mary K. Mulcahey, Anthony Deldin

**Affiliations:** 1 Department of Orthopaedic Surgery and Rehabilitation, Loyola University Chicago Stritch School of Medicine, Maywood, IL, United States; 2 Applied Health Science, Loyola University Chicago, Chicago, IL, United States

**Keywords:** countermovement jump (CMJ), inter-limb asymmetry, rate of force development (RFD), sex differences, student athletes, volleyball

## Abstract

**Objective:**

To compare jump performance and inter-limb force asymmetry between male and female collegiate volleyball athletes, examine short-term in-season stability and identify which asymmetry metrics best support applied monitoring and reconditioning decisions.

**Methods:**

In a prospective cohort, NCAA Division I volleyball athletes (23 males, 18 females) completed weekly bilateral countermovement jumps (CMJ). Inter-limb asymmetry indices for peak force, braking force, and rate of force development (RFD) were computed as the absolute percentage difference between limbs (100·|Right−Left|/max[Right, Left]) and summarized using 5-week centered rolling means. Between-sex comparisons used two-tailed tests with Benjamini–Hochberg false discovery rate control. Athlete profiles were derived via k-means clustering on the three asymmetry features, and a shallow decision tree identified the most informative metric.

**Results:**

Males produced higher absolute jump outputs, whereas inter-limb asymmetry magnitudes were modest (approximately 5%–11%) and overlapped substantially between sexes; no between-sex differences in peak-force, braking-force, or RFD asymmetry remained significant after correction (all q = 0.207). A *post hoc* power analysis indicated that the observed RFD asymmetry effect (d = 0.40) had limited power, so moderate sex differences cannot be excluded. Group trajectories of asymmetry were relatively stable across the competitive window. Clusters derived from asymmetry features were distinct, and the decision tree consistently prioritized RFD asymmetry as the primary feature distinguishing asymmetry-based profiles, with peak force asymmetry secondary and braking asymmetry less discriminative.

**Conclusions:**

In-season CMJ force asymmetry in collegiate volleyball was small, largely overlapping between sexes, and showed limited systematic drift at the group level. RFD asymmetry emerged as the primary descriptor of asymmetry-based profiles but should be interpreted cautiously alongside more stable CMJ outputs. Summarizing weekly asymmetry with 5-week centered rolling means provides coach-ready signals for athlete-specific, profile-based monitoring rather than sex-based thresholds and offers guidance for structuring asymmetry-based monitoring and prioritizing training and reconditioning emphases.

## Introduction

Successful volleyball performance hinges on explosive verticality, where the ability to generate rapid force during takeoff and dissipate kinetic energy upon landing defines neuromuscular readiness for repeated jump and landing demands (1, 2). Performance relies on repeated explosive jumping in offensive and defensive actions. Offensively, the hit (spike/attack/kill) requires leaping and striking the ball at maximal reach, while defensively front-row players jump at the net to block. During each jump-landing sequence, the landing phase must dissipate the kinetic energy generated during takeoff (1, 2). Players need rapid force and power generation to achieve vertical height, and safe, controlled landings to reduce undue joint loading and maintain readiness for subsequent plays. Accordingly, vertical jump assessments are widely used to evaluate neuromuscular readiness for jump performance, monitor training adaptations, and identify biomechanical risk factors for lower-extremity injury (3, 4). Because these jump and landing demands place substantial loads on the lower extremities, force-plate–derived biomechanical metrics, such as peak and braking forces, rate of force development (RFD), and inter-limb symmetry indices, are increasingly emphasized as markers of neuromuscular efficiency and lower-extremity joint loading patterns (5, 6).

Sex differences in jump biomechanics are well documented: males typically outperform females in jump height, peak force, and reactive strength, largely attributable to greater skeletal muscle mass and tendon stiffness (7, 8). However, sex-based comparisons of neuromuscular timing, landing strategies, and RFD have produced more mixed findings (9, 10). Some evidence suggests that normalized explosive strength parameters may not differ meaningfully between male and female athletes when adjusted for body mass or training experience (5, 11). Despite these physiological differences, limited research has examined whether asymmetries in jump force or timing also differ by sex in elite volleyball players.

Inter-limb asymmetry has emerged as a key biomechanical marker in sport performance and injury research. Vertical jump asymmetries of approximately 10%–15% have been commonly reported in jumping athletes, with wide variability between individuals (12, 13). Prospective studies have linked certain biomechanical deficits and the magnitude of asymmetry with elevated lower limb injury risk (14, 15). However, asymmetry often varies substantially between individuals and may not align with traditional demographic variables like sex or training level (16, 17). The interpretation of these data is highly sensitive to computation choices and metric dependence (13, 17). Moreover, some compensatory patterns, particularly unilateral deficits in force rate rather than magnitude, may go undetected in standard performance metrics (5, 11). Longitudinal data suggest that while absolute performance fluctuates, asymmetry profiles may remain relatively stable across a competitive season, providing a consistent baseline for monitoring (18).

Accordingly, this study compared jump performance and inter-limb force asymmetry between male and female NCAA Division I volleyball athletes, examined short-term in-season trends, and identified asymmetry profiles via unsupervised learning. We hypothesized that (1) males would demonstrate higher absolute outputs but similar proportional asymmetry, (2) in-season values would be relatively stable when summarized using 5-week centered rolling means, and (3) RFD asymmetry would be the most informative triage metric for applied monitoring. Although males typically demonstrate greater absolute jump outputs, existing literature suggests that inter-limb asymmetry may depend more on individual movement strategies, task characteristics, and calculation approach than on sex alone (16, 17). Because deficits in the rate of force generation may reveal unilateral compensatory strategies that are not fully captured by force-magnitude variables alone, RFD asymmetry may provide particularly sensitive information for athlete monitoring (5, 11). By identifying distinct asymmetry profiles through unsupervised learning, this study aims to provide practitioners with a data-driven framework for individualized monitoring and targeted reconditioning in collegiate volleyball.

## Methods

### Subjects

This study used a prospective observational design during routine in-season monitoring of NCAA Division I volleyball athletes from August through November 2023. Data were collected from 41 athletes (23 males, 18 females), all of whom were active roster members participating fully in team activities and cleared for unrestricted participation by the medical and performance staff. Where recorded, male athletes were 21.2 ± 1.2 years of age, 195.8 ± 5.1 cm tall, and 83.1 ± 8.8 kg in body mass, and female athletes were 20.5 ± 0.7 years of age, 176.1 ± 7.2 cm tall, and 68.0 ± 8.0 kg in body mass. During the monitoring period, athletes trained as part of their normal team program, which included regular court-based practices and supervised resistance training led by the strength and conditioning staff; training volume varied across the period in accordance with the team’s schedule. Inclusion criteria were (1) active roster status during the target season, (2) medical clearance for full participation in training and competition, and (3) participation in the team’s routine pre-practice CMJ monitoring sessions. No athletes were excluded *a priori* on the basis of surgical history or prior injury. All healthy, rostered athletes were eligible, and the only participants withheld from testing were those medically restricted by the athletic training staff. Trials were excluded only if they were unusable because of protocol deviations, including failure to land with both feet on the force plates or failure to return to a stable standing position after landing. All data were de-identified before analysis. This retrospective analysis of de-identified data collected during routine, pre-practice force plate testing was approved by the Institutional Review Board at our institution (IRB Application Number: 10608; approval date: January 26, 2026). The IRB determined the study to be minimal risk and granted a waiver of written informed consent in accordance with applicable regulations.

### Countermovement vertical jump

Athletes performed countermovement jump (CMJ) assessments during weekly scheduled performance monitoring sessions. Before testing, each athlete completed a self-selected dynamic warmup lasting approximately 3–5 min, typically including bodyweight squats, toe touches, hopping, or stationary cycling. Following the warm-up, athletes performed three maximal-effort bilateral CMJs. Each jump was initiated from an upright standing position with one foot placed on each of two force platforms. Participants were instructed to keep their hands on their hips throughout the movement to eliminate arm swing and isolate lower-extremity power production. They were instructed to descend as quickly as possible to a self-selected depth and then immediately jump vertically as high as possible, returning to a stable standing position upon landing (6).

All vertical ground reaction force data were collected using dual force plates (Bertec Corporation, Columbus, OH, USA) sampling at 1500 Hz and were acquired using the Noraxon MyoForce Research Pro software system (Noraxon USA Inc., Scottsdale, AZ, USA). Testing was administered by the team’s strength and conditioning staff in collaboration with the research team, who supervised protocol adherence; any trial with visible deviations was discarded. A rest interval of 15–30 s was provided between jumps to reduce the influence of fatigue.

### Signal processing and outcome variables

Force-time data from each valid CMJ trial were exported and processed in Python (v3.10). For primary analyses, performance metrics were averaged across valid trials and sessions to generate per-athlete mean values. Force onset was defined as the point at which vertical force exceeded quiet-standing baseline by 5 standard deviations, a conservative, pre-specified laboratory threshold used to minimize false detections due to baseline noise in high-frequency force-plate signals; landing was identified as the first force rise greater than 20 N above post-flight baseline. The braking phase was defined as the interval from force onset to minimum center-of-mass velocity, which marked the transition from braking to propulsion and was determined from integrated net vertical force using the impulse-momentum approach. The propulsion phase was defined from minimum center-of-mass velocity to takeoff, with takeoff identified when vertical ground reaction force fell below 20 N. These 20 N thresholds represent a small absolute force level commonly used to distinguish airborne phases from low-amplitude noise in force-plate recordings (27).

The primary force-based variables of interest were peak takeoff force (N), braking force (N), and RFD (N·s^−1^). Peak force was defined as the maximum vertical force recorded before takeoff. Braking force was defined as the mean vertical force across the braking phase. RFD was defined as the peak slope of the force-time curve within the 0–200 ms window after force onset. Jump height (cm) was calculated from flight time using the software-derived force plate output. An impulse-momentum-derived estimate of jump height was also examined in sensitivity analyses, with no meaningful change in study conclusions. Because flight time and jump height are highly collinear, flight time was not included in inferential analyses and was retained only for internal quality control. RSImod was calculated as jump height (impulse-momentum-derived; m) divided by time to take-off from force onset to takeoff (s), yielding RSImod in m·s⁻¹. In addition to peak takeoff force, braking force, and RFD, the following CMJ performance variables were derived for descriptive and inferential analyses: peak landing force (N; maximal vertical force recorded during landing), takeoff velocity (m·s⁻¹; vertical velocity at takeoff), and net impulse (N·s; time-integral of net vertical force over the propulsion phase). These variables are summarized in Table 1.

**Table 1:** Sex-based comparison of CMJ performance metrics.

Metric	Test used	Male (mean ± SD)	Female (mean ± SD)	Normality *p* (M/F)	*p*-value	Effect size	q-value
Peak takeoff force	t-test	2,050.9 ± 216.7	1,710.2 ± 253.9	0.455/0.678	6.81E-05	1.44	0.0001
Peak landing force	t-test	1,917.2 ± 218.2	1,647.4 ± 258.6	0.409/0.994	0.00118	1.13	0.0014
Takeoff velocity	Mann–Whitney U	2.69 ± 0.24	2.38 ± 0.22	0.029/0.912	0.00011	0.86	0.0002
Jump height	Mann–Whitney U	39.81 ± 7.43	30.51 ± 5.44	0.012/0.550	4.93E-05	0.87	0.0001
RSImod	t-test	0.45 ± 0.07	0.37 ± 0.06	0.262/0.245	0.00044	1.2	0.0006
RFD	Mann–Whitney U	10,023.5 ± 2,056.1	9,279.2 ± 3,161.5	0.164/0.008	0.09018	0.66	0.0902
Net impulse	t-test	225.48 ± 23.05	175.86 ± 17.55	0.449/0.588	1.63E-09	2.42	<0.0001

M, male; F, female; RSImod, modified reactive strength index; RFD, rate of force development. *P*-value significance: *p* < 0.05.

To contextualize measurement error, between-session reliability was evaluated in a subset of athletes with repeated testing sessions. Peak force, jump height, and RSImod demonstrated good-to-excellent reliability, with intraclass correlation coefficients in the good-to-excellent range and coefficients of variation below 10%, whereas asymmetry indices, particularly RFD asymmetry, showed higher within-athlete variability. These estimates informed interpretation of small longitudinal changes.

### Inter-limb asymmetry and longitudinal profiling

Limb-specific force values were obtained from the individual force plates. Inter-limb asymmetry (%) for peak force, braking force, and RFD was calculated as the absolute difference between limbs divided by the larger limb value and multiplied by 100, such that asymmetry = |Right−Left|/max[Right, Left] × 100. This approach bounded values between 0 and 100 and minimized instability associated with small denominators. A secondary sensitivity analysis using the mean of the two limbs in the denominator was also performed to evaluate formula dependence, and results were qualitatively unchanged. Absolute rather than directional asymmetry values were used for primary analyses to provide a bounded, easily interpretable index for applied monitoring and to facilitate profile-based comparisons when small imbalances may change direction over time, with the acknowledgment that this approach does not retain information about which limb is stronger or weaker.

To characterize short-term in-season trends, weekly per-athlete CMJ data were summarized using 5-week centered rolling means. This approach was selected to reduce week-to-week noise and provide more stable decision signals for applied monitoring. Group-level trajectories were then described across the competitive window. To identify athlete-specific asymmetry presentations, k-means clustering was performed using peak force asymmetry, braking force asymmetry, and RFD asymmetry. The number of clusters was selected using elbow-plot and silhouette diagnostics, with preference given to clinically interpretable solutions. To improve interpretability of cluster membership, a shallow decision tree with a maximum depth of 2 was subsequently trained to identify the asymmetry feature most responsible for separating athlete profiles.

### Statistical analysis

All statistical analyses were performed in Python (v3.10) using standard scientific libraries, including scipy and statsmodels for inferential testing and scikit-learn for clustering and classification. Distributional assumptions were evaluated with the Shapiro–Wilk test. Between-sex comparisons were performed using independent-samples t-tests when normality assumptions were met and Mann–Whitney U tests otherwise. All tests were two-tailed with statistical significance set at *α* = 0.05. To account for multiple comparisons within related families of tests, *p* values were adjusted using the Benjamini–Hochberg false discovery rate procedure with q = 0.05. Effect sizes were reported as Hedges’ g for parametric comparisons and rank-biserial correlations for nonparametric comparisons, with 95% confidence intervals where appropriate. In accordance with reporting conventions, *p* values are reported to three decimal places, with values smaller than 0.001 reported as *p* < 0.001. Analyses were conducted on complete cases after quality-control screening. Sample size was determined by the fixed roster of the participating teams rather than an *a priori* power calculation; a *post hoc* power analysis for the primary between-sex comparison in RFD asymmetry indicated that the available sample provided limited power to detect moderate effect sizes. This observational study was reported in accordance with the STROBE guidelines and EQUATOR Network recommendations.

## Results

### Sex-based comparisons of CMJ performance

Male athletes outperformed females on most CMJ performance metrics after false discovery rate (FDR) correction. Significant between-sex differences favored males for peak takeoff force, peak landing force, jump height, takeoff velocity, RSImod, and net impulse (all q < 0.01). The largest effects were observed for net impulse (*p* = 1.63 × 10⁻⁹, d = 2.42) and peak takeoff force (*p* = 6.81 × 10⁻⁵, d = 1.44), with RSImod also markedly higher in males (*p* = 0.00044, d = 1.20). RFD trended higher in males but did not remain statistically significant after correction (*p* = 0.090, d = 0.66). Full descriptive statistics and between-sex comparisons for CMJ performance variables are reported in Table 1. The sex-specific violin plots for performance variables are provided in Supplementary Figure S2 to avoid redundancy with Table 1. Because flight time and takeoff velocity are highly collinear with jump height, jump height is emphasized as the primary displacement outcome; flight time and takeoff velocity are retained only for transparency where reported.

### Inter-limb asymmetry by sex

**Figure 1:**
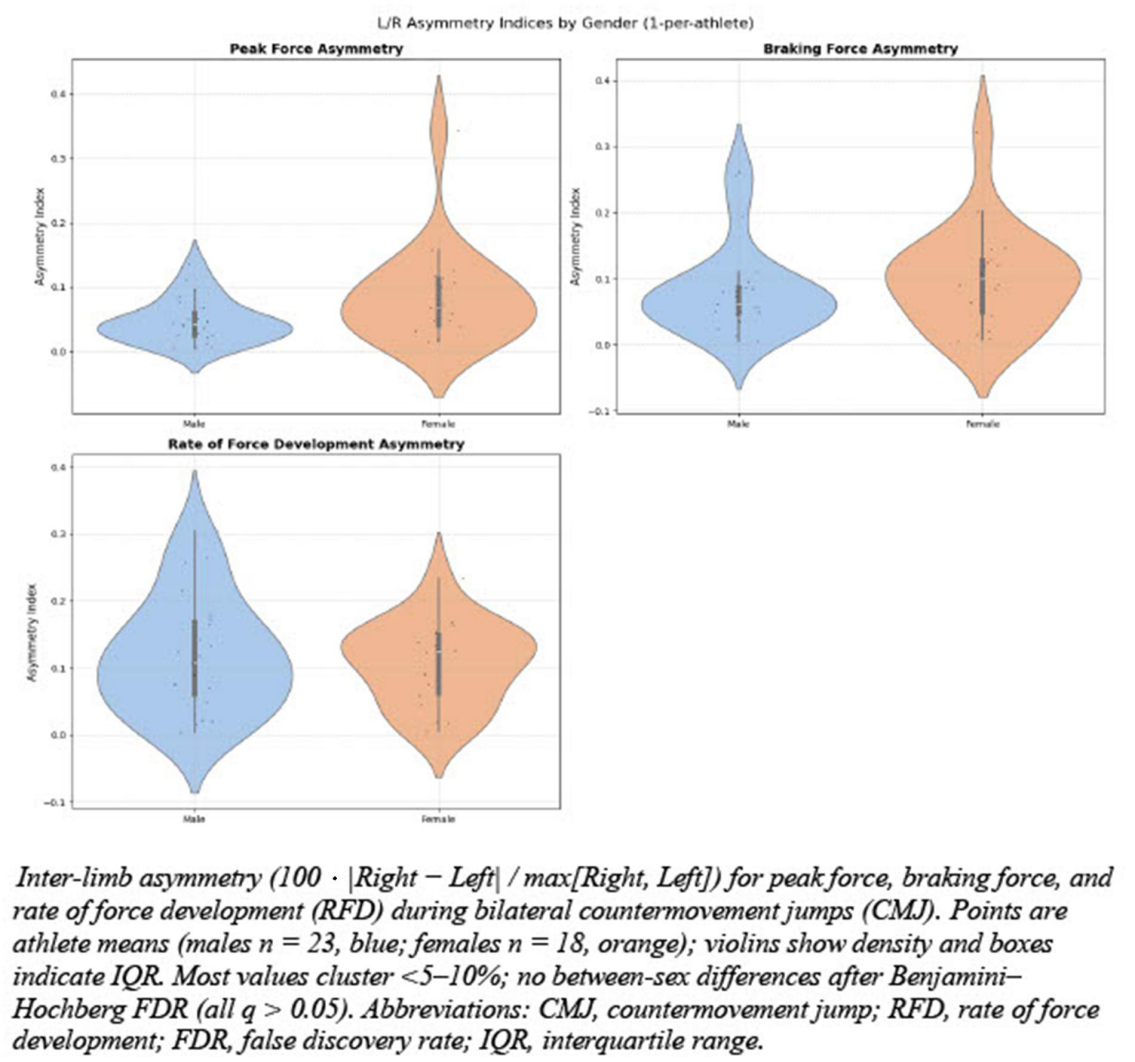
Sex-based comparisons of jump force asymmetry indices.

Asymmetry indices for peak force, braking force, and RFD did not differ significantly between sexes after multiple-comparison control (all q = 0.207). On average, females exhibited somewhat greater peak-force and braking-force asymmetry, whereas males showed somewhat greater RFD asymmetry; however, effects were small to moderate in magnitude and overlapped substantially between sexes. Specifically, peak-force asymmetry was 5.24 ± 3.07% in males and 8.68 ± 7.54% in females (d = −0.63), braking-force asymmetry was 8.78 ± 6.37% in males and 10.79 ± 7.11% in females (d = −0.30), and RFD asymmetry was 15.11 ± 5.98% in males and 12.92 ± 4.63% in females (d = 0.40; all q = 0.207). These results indicate that, within the limits of the available sample size, athlete sex did not exert a systematic influence on inter-limb asymmetry in this cohort. Descriptive statistics and inferential results for asymmetry metrics are summarized in Table 2, and sex-specific distributions are illustrated in Figure 1.

**Table 2:** Sex-based comparison of inter-limb asymmetry metrics.

Metric	Male mean ± SD (%)	Female mean ± SD (%)	*p*-value	Effect size (d)	q-value
Peak-force asymmetry	5.24 ± 3.07	8.68 ± 7.54	0.095	−0.63	0.207
Braking-force asymmetry	8.78 ± 6.37	10.79 ± 7.11	0.145	−0.30	0.207
RFD asymmetry	15.11 ± 5.98	12.92 ± 4.63	0.207	0.40	0.207

Values are athlete-level means ± SD using the max-denominator asymmetry formula: 100   ×   |Right−Left|/max(Right, Left). Effect size is Cohen’s d calculated as male minus female. q-values are Benjamini–Hochberg FDR-adjusted across the three asymmetry comparisons.

### Longitudinal trends across the competitive window

Across early, mid, and late season (August 1–November 1), no significant within-group changes were detected for CMJ performance or asymmetry metrics after FDR correction (all q > 0.05). Weekly trajectories summarized with 5-week centered rolling means and standard errors of the mean are shown in Supplementary Figure S1, illustrating the relative stability of group-level performance and asymmetry signals during the competitive window.

### Asymmetry profiles and decision-tree classification

**Figure 2:**
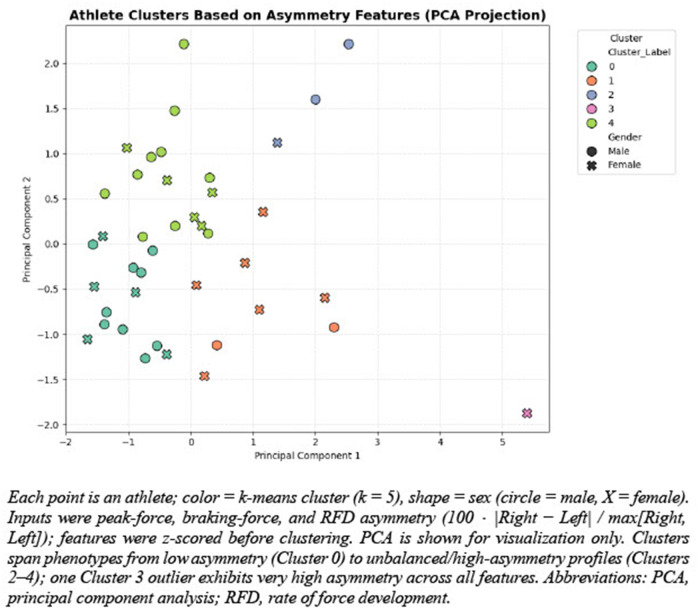
Athlete clusters based on asymmetry features (PCA projection).

**Figure 3:**
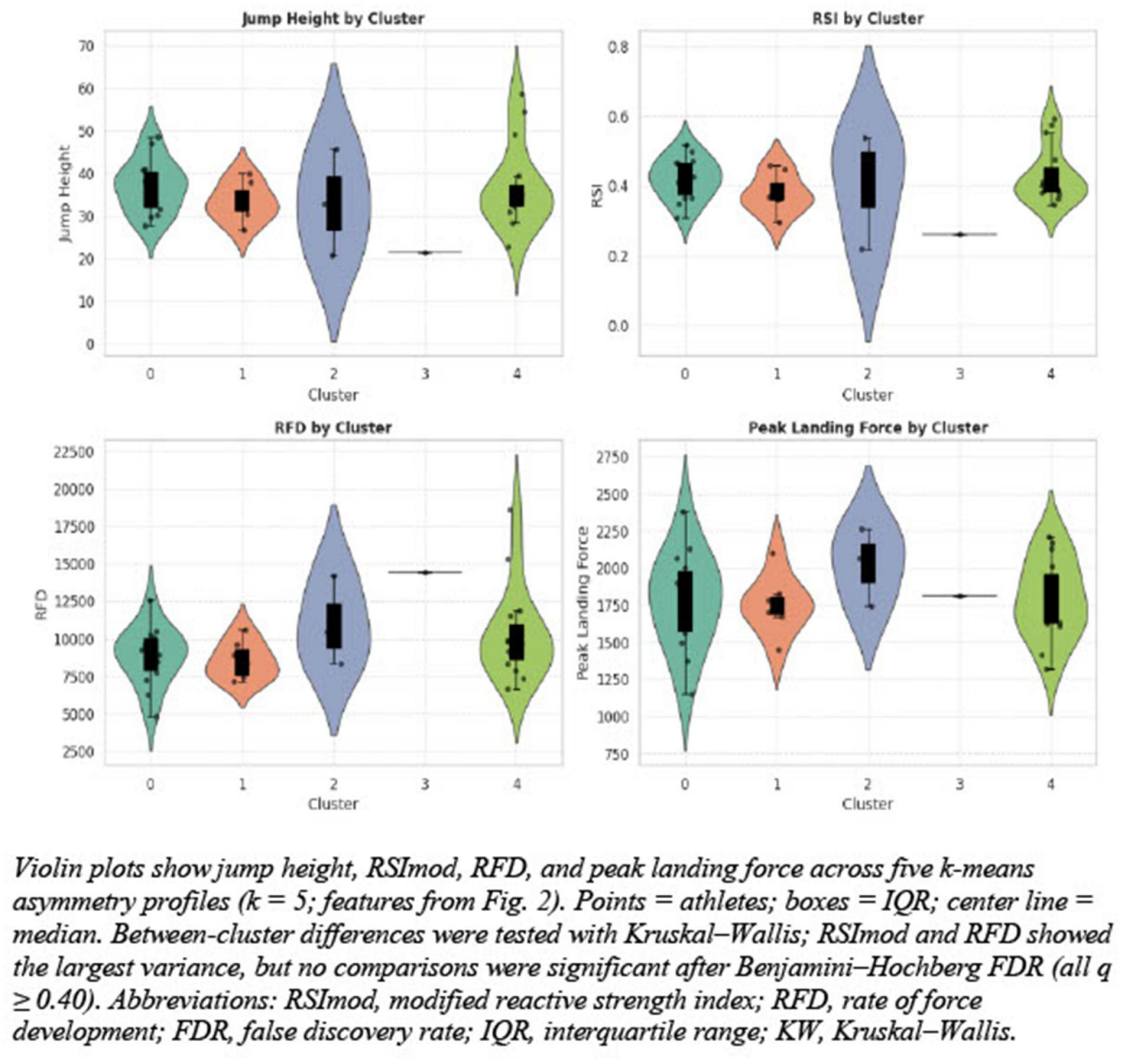
Performance metrics by asymmetry cluster.

Unsupervised clustering of asymmetry features (peak force asymmetry, braking force asymmetry, and RFD asymmetry) revealed distinct athlete profiles (Figure 2). A shallow decision tree (maximum depth = 2) identified RFD asymmetry as the primary split and most informative variable for profile discrimination in this cohort, with peak force asymmetry contributing secondarily and braking asymmetry less discriminative (Figure 3). To evaluate whether these profiles reflected broader performance characteristics, jump height, RSImod, RFD, and peak landing force were compared across clusters using the Kruskal–Wallis test. RSImod and RFD showed the largest between-cluster variance (statistic = 6.00 and 6.48, respectively), but none of the performance comparisons reached statistical significance after FDR correction (all q ≥ 0.40), indicating that asymmetry-based profiles captured differences in inter-limb force strategy that were not reflected in conventional CMJ output measures.

## Discussion

Males produced higher countermovement-jump outputs across most performance metrics (seven of eight, including peak force, jump height, and net impulse), whereas inter-limb asymmetry magnitudes were comparable between sexes and generally small (<5%–10%) (10). RFD trended higher in males but did not survive FDR correction. Together with small between-sex effect sizes, these findings support the view that CMJ asymmetry is primarily an individual characteristic rather than sex-specific in collegiate volleyball, consistent with prior syntheses linking asymmetry to athlete-level variability rather than biological sex *per se* (16). Practically, programming decisions should be guided by each athlete’s asymmetry profile rather than sex.

Across the August–November window, no statistically significant within-group changes were observed for performance or asymmetry after multiple-comparison control (all q > 0.05), and weekly trajectories were relatively stable (18). We visualized weekly data as 5-week centered rolling means with SEM to align with in-season monitoring practice and reduce the influence of week-to-week measurement noise (19). Qualitative inspection suggested small reductions in braking and RFD asymmetry among female athletes, but these trends were not statistically significant and warrant prospective evaluation.

Interpretation of asymmetry magnitudes depends on both task and computation. Unilateral and bilateral tasks can yield different absolute values, and alternative formulae can shift magnitudes; therefore, comparisons should be made within a consistent task and calculation framework to avoid misclassification (13, 16, 20). In this study, we standardized a bilateral CMJ task and a single primary asymmetry formula, defined as the absolute difference between limbs divided by the larger limb value, to bound values between 0 and 100 and reduce instability associated with small denominators. This choice facilitates longitudinal comparability and applied interpretation but, as a trade-off, does not retain information about the direction of the asymmetry or side-specific dominance. A secondary analysis using the mean of both limbs in the denominator produced qualitatively similar conclusions, suggesting that the main findings were not driven by the specific denominator. However, future work should incorporate directional asymmetry and participant-level descriptive trajectories to better characterize fluctuations in both the magnitude and direction of asymmetry across individuals. Because RFD is sensitive to methodological choices, we followed established recommendations for event detection and windowing when deriving RFD from force-time data (5, 6, 11).

Unsupervised clustering of peak force, braking force, and RFD asymmetry revealed distinct athlete profiles (21), and a depth-constrained decision tree identified RFD asymmetry as the primary split and most informative screening feature, with peak force asymmetry secondary and braking asymmetry less discriminative. This taxonomy provides interpretable profiles that map onto targeted monitoring and training emphases (4). While traditional performance metrics differentiated males and females on absolute outputs, they did not capture heterogeneity in inter-limb force strategies; asymmetry profiling offers complementary information for individualization that is not apparent from jump height or impulse alone (21). Where tested, performance metrics did not differ across clusters after multiple-comparison control (all q > 0.05), reinforcing the value of asymmetry features for individualized monitoring rather than as proxies for jump output.

The overall magnitude of asymmetry (<5%–10% for most athletes) is consistent with ranges reported in trained populations and lies at the lower end of the commonly cited 10%–15% bands in jumping athletes (13, 22). Although larger inter-limb differences have been associated with elevated injury risk in prior prospective work (14, 15), asymmetry thresholds are task- and metric-specific and should not be applied universally. Because injury outcomes were not collected here, causal inferences are not possible; asymmetry signals should be interpreted alongside workload exposure, readiness/wellness indicators, and movement-quality assessments to reduce false positives (4). In addition, morphological asymmetries in limb size or segmental lean mass, which were not assessed in this study, may interact with functional force asymmetry and merit consideration in future work (23, 24). Training age and detailed training-history descriptors were not systematically recorded and could not be reported; future studies should capture these variables to aid replication and interpretation.

From a programming standpoint, relatively stable in-season trajectories suggest that monitoring is best positioned for early detection of meaningful shifts and short, targeted adjustments, whereas larger symmetry changes are more realistically pursued during off-season blocks. Previous work has shown that both the magnitude and direction of inter-limb asymmetry can vary across a season and that stable directional biases may carry side-specific information relevant to screening and rehabilitation (25, 26). In the present cohort, small RFD asymmetries frequently changed direction between sessions, reinforcing the decision to treat absolute RFD asymmetry as a noisy signal at low magnitudes and to interpret it alongside more reliable CMJ outputs. Practical translation is summarized in the Practical Applications section: use sex-neutral thresholds, prioritize RFD asymmetry within a broader profile-based framework, establish athlete-specific baselines via weekly dual-plate CMJs summarized with 5-week centered rolling means, and apply profile-based programming for balanced, braking-dominant, RFD-dominant, and combined presentations (4, 5, 11, 16).

## Practical applications

Clinicians and coaches working with similar collegiate volleyball populations may consider RFD asymmetry as a first-line screening feature, interpreted alongside more stable CMJ outputs, and apply sex-neutral thresholds when interpreting weekly dual-plate CMJs. These recommendations are cohort- and task-specific and should not be assumed to generalize to other sports or testing configurations without sport- and metric-specific evaluation. Establish athlete-specific baselines over 3–5 weeks, then summarize in-season data with a 5-week centered rolling mean to stabilize decisions. Use tiered actions: Green (<5% and <5% from baseline) indicates maintenance; Yellow (5%–10% or ≥5% above baseline for 1 week) prompts unilateral power work and tempo eccentrics with re-check in 1–2 weeks; Red (>10% or ≥5% above baseline for ≥2 weeks) warrants reducing high-impact bilateral plyometrics and emphasizing unilateral power plus eccentric control, with sports-medicine review if persistent. Tailor by profile: maintain for Balanced; add tempo eccentrics, landing circuits, and split-squat isometrics for Braking-dominant; prioritize unilateral jumps and velocity-targeted jump squats for RFD-dominant; and blend both approaches while reducing chaotic change-of-direction and high-impact plyometrics by approximately 20%–30% for combined profiles. See Supplementary Figure S3 (Asymmetry Action Card) for the visual decision pathway. These tiered actions and profile-based prescriptions are intended as decision-support guidelines grounded in the observed distributions and existing asymmetry literature (4, 13, 16–18, 20, 21) and should be refined prospectively as data on training responses and injury outcomes become available. These bands are metric- and task-dependent and are applied here to CMJ force and RFD asymmetry in this cohort as hypothesis-generating guidance, in line with prior work showing that asymmetry thresholds vary by variable and context (e.g., Menzel et al. (28)), and should be refined as prospective outcome data become available.

## Data Availability

The raw data supporting the conclusions of this article will be made available by the authors, without undue reservation.

## Supplementary material

Supplementary Figure S1 Weekly biomechanical trends by sex (August 1–November 1). Mean weekly trajectories (±1 SEM) for key performance and asymmetry metrics in male (blue) and female (orange/red) athletes. Data grouped by calendar week and smoothed with a 5-week centered rolling mean. Metrics: jump height, RSImod, RFD, peak landing force, and three inter-limb asymmetry indices (peak-force, braking-force, RFD). Visual trends show relative stability; no within-group changes were statistically significant after multiple-comparison control (all q > 0.05). Abbreviations: RSImod, modified reactive strength index; RFD, rate of force development; SEM, standard error of the mean.

Supplementary Figure S2 Sex-based performance comparisons (violin plots). Violin plots of CMJ performance metrics (e.g., jump height, net impulse, takeoff velocity, RSImod) for male (blue) and female (orange) athletes. Each point represents an athlete-level in-season mean; violins depict density, boxes indicate IQR, and center lines mark medians. Males exhibited higher outputs across most metrics; exact p-values and effect sizes are reported in the Results and Table 1. Non-significant results are indicated as q > 0.05. Abbreviations: CMJ, countermovement jump; RSImod, modified reactive strength index; IQR, interquartile range; FDR, false discovery rate.

Supplementary Figure S3 Asymmetry Action Card (collegiate volleyball). Visual decision pathway for profile-based, sex-neutral monitoring using weekly dual-plate CMJs summarized with a 5-week centered rolling mean. Summarizes screening focus (prioritize RFD asymmetry, peak-force secondary), baseline/cadence guidance, tiered thresholds (Green <5%; Yellow 5–10%; Red >10%, or ≥5% above personal baseline), and profile-based prescriptions for balanced, braking-dominant, RFD-dominant, and combined presentations. Intended as hypothesis-generating decision support, not diagnostic thresholds. Abbreviations: CMJ, countermovement jump; RFD, rate of force development; COD, change of direction.
