# Supplementary material for: Athlete-specific force asymmetry monitoring in collegiate volleyball: a prospective observational cohort
Source: Front Sports Act Living. 2026 Aug 4;8:1885824. doi: 10.3389/fspor.2026.1885824 (PMC13481779; doi:10.3389/fspor.2026.1885824)
Supplement: Supplementary file 1 [file Supplementaryfile3.pdf]

Asymmetry Action Card — Collegiate Volleyball

Use weekly dual-plate CMJs; summarize with a 5-week centered rolling mean. Apply sex-neutral thresholds.

Screening Focus

- Prioritize RFD asymmetry; track peak-force secondarily
- Apply sex-neutral thresholds for all athletes
- When time-constrained, triage with RFD only

Green Threshold

- < 5% AND < 5% from personal baseline
- Action: maintain plan; progress normally

Yellow Threshold

- 5–10% OR ≥ 5% above baseline for 1 week
- Action: add unilateral power + tempo eccentrics; re-check 1–2 wks

Red Threshold

- > 10% OR ≥ 5% above baseline for ≥ 2 consecutive weeks
- Action: reduce high-impact bilateral plyos 20–30%; emphasize unilateral power + eccentric control; consider sports-med review if persistent

Baselines & Cadence

- Baseline: 3–5 weeks of weekly CMJs (≥3 trials)
- In-season: test weekly; compute 5-week centered rolling mean
- Store athlete-specific thresholds for decision consistency

Profile-Based Prescriptions & Return-to-Normal

- Balanced → maintain bilateral progressions; monitor bi-weekly
- Braking-dominant → tempo eccentrics (3–4 s), landing mechanics circuit, split-squat isometrics
- RFD-dominant → unilateral jumps; assisted/light-load jump squats with velocity targets; contrast pairings
- Combined → blend eccentric control + unilateral power; reduce chaotic COD/high-impact bilateral plyos 20–30% for 1–2 weeks
- Return to normal: require two consecutive improvements (flags) before restoring full plyometric density
