# Supplementary material for: Athlete-specific force asymmetry monitoring in collegiate volleyball: a prospective observational cohort
Source: Front Sports Act Living. 2026 Aug 4;8:1885824. doi: 10.3389/fspor.2026.1885824 (PMC13481779; doi:10.3389/fspor.2026.1885824)
Supplement: Supplementary file 2 [file Supplementaryfile1.docx]

Supplementary Figure S1. Weekly biomechanical trends by sex (August 1–November 1)

*Mean weekly trajectories (±1 SEM) for key performance and asymmetry metrics in male (blue) and female (orange/red) athletes. Data grouped by calendar week and smoothed with a 5-week centered rolling mean. Metrics: jump height, RSImod, RFD, peak landing force, and three inter-limb asymmetry indices (peak-force, braking-force, RFD). Visual trends show relative stability; no within-group changes were statistically significant after multiple-comparison control (all q > 0.05). Abbreviations: RSImod, modified reactive strength index; RFD, rate of force development; SEM, standard error of the mean.*
