# Supplementary material for: Athlete-specific force asymmetry monitoring in collegiate volleyball: a prospective observational cohort
Source: Front Sports Act Living. 2026 Aug 4;8:1885824. doi: 10.3389/fspor.2026.1885824 (PMC13481779; doi:10.3389/fspor.2026.1885824)
Supplement: Supplementary file 3 [file Supplementaryfile2.docx]

Supplementary Figure S2. Sex-based performance comparisons (violin plots)

*Violin plots of CMJ performance metrics (e.g., jump height, net impulse, takeoff velocity, RSImod) for male (blue) and female (orange) athletes. Each point represents an athlete-level in-season mean; violins depict density, boxes indicate IQR, and center lines mark medians. Males exhibited higher outputs across most metrics; exact p-values and effect sizes reported in Results. Differences surviving FDR are noted in-text; non-significant results indicated as q > 0.05. Abbreviations: CMJ, countermovement jump; RSImod, modified reactive strength index; IQR, interquartile range; FDR, false discovery rate.*
